# Ferromagnetic glass-coated microwires with good heating properties for magnetic hyperthermia

**DOI:** 10.1038/srep39300

**Published:** 2016-12-19

**Authors:** A. Talaat, J. Alonso, V. Zhukova, E. Garaio, J. A. García, H. Srikanth, M. H. Phan, A. Zhukov

**Affiliations:** 1 Department of Physics, University of South Florida, Tampa, FL 33620, USA; 2 Dpto. Física de Materiales, UPV/EHU, 20018 San Sebastián, Spain; 3 Dpto. de Física Aplicada, EUPDS, UPV/EHU, 20018, San Sebastián, Spain; 4 BCMaterials Edificio N°. 500, Parque Tecnológico de Vizcaya, 48160, Derio, Bilbao, Spain; 5 Department of Electricity and Electronics, University of Basque Country, Leioa 48940, Spain; 6 Department of Applied Physics II, University of Basque Country, Leioa 48940, Spain; 7 IKERBASQUE, Basque Foundation for Science, 48011 Bilbao, Spain

## Abstract

The heating properties of Fe_71.7_Si_11_B_13.4_Nb_3_Ni_0.9_ amorphous glass-coated microwires are explored for prospective applications in magnetic hyperthermia. We show that a single 5 mm long wire is able to produce a sufficient amount of heat, with the specific loss power (SLP) reaching a value as high as 521 W/g for an AC field of 700 Oe and a frequency of 310 kHz. The large SLP is attributed to the rectangular hysteresis loop resulting from a peculiar domain structure of the microwire. For an array of parallel microwires, we have observed an SLP improvement by one order of magnitude; 950 W/g for an AC field of 700 Oe. The magnetostatic interaction strength essential in the array of wires can be manipulated by varying the distance between the wires, showing a decreasing trend in SLP with increasing wire separation. The largest SLP is obtained when the wires are aligned along the direction of the AC field. The origin of the large SLP and relevant heating mechanisms are discussed.

Magnetic hyperthermia is one of the most promising techniques for localized cancer treatment^1^,^2^. This technique is based on heating the tumor area as a consequence of the application of an alternating magnetic field to a magnetic material. By raising the temperature in a localized way inside the tumor area up to 40–45 °C, the cancer cells are irreversibly damaged while the surrounding tissue remains almost unaffected^3^. The heating efficiency of a magnetic material under an alternating magnetic field is often assessed by the Specific Absorption Rate (SAR), although the term Specific Loss Power (SLP) is more appropriate for magnetic materials. In order to reduce the dosage needed for hyperthermia treatment while using applied field and frequency values below the safety and patient tolerance limits^4^, it is essential to increase the SLP as much as possible. From a theoretical point of view, the heating capacity of a magnetic material can be related to hysteresis losses and/or eddy currents^2^. Hysteresis losses are related to the magnetic domain orientation and can be represented by the hysteresis loop area of the material, being the SLP = *A*∙*f*, where *A* is the area of the AC hysteresis loop and *f* the exciting frequency of the magnetic field. Eddy currents are generated by electromagnetic induction and lead to Joule heating of the material. Different materials often have different optimal sizes for achieving maximum SLP, and for *in-vivo* applications the employed material must be biocompatible.

Magnetic hyperthermia has mostly relied on iron oxide nanoparticles, since the nanoparticles are biocompatible, they have the appropriate size to interact with cancer cells and can be guided toward the tumor site^1^,^2^. However, these nanoparticles possess a series of limitations (relatively low saturation magnetization, tendency to aggregate, and moderate heating efficiency) that restrict their clinical realization^5^,^6^. Moreover, when the particles are injected they tend to be covered with proteins, encouraging their phagocytosis^7^. They can also be recognized as “foreign bodies” and eliminated by the reticuloendothelial system^8^,^9^. And once the treatment is finished, the nanoparticles tend to accumulate in the liver and spleen, which could lead to toxic side effects^10^,^11^. To avoid these problems, alternative magnetic hyperthermia materials have been explored^12^,^13^,^14^,^15^,^16^,^17^. For instance, the use of magnetic fibers or wires for minimally invasive hyperthermia has recently been proposed^13^,^15^.

To this respect, soft ferromagnetic amorphous glass-coated microwires (AGCMs) prepared by the modified Taylor-Ulitovsky technique are very promising. The AGCMs present outstanding magnetic properties (e.g. magnetic bistability, giant magneto-impedance effect (GMI), fast domain wall propagation) for a variety of applications in magnetic sensors, microelectronics, security, and biomedical sensors^18^,^19^,^20^,^21^. The capacity of producing long and continuous wires with reduced dimension (diameter, 0.5–100 μm) and circular symmetry yields a significant advantage over other materials. Moreover, the glass coating itself provides enhanced biocompatibility. Fe-based AGCMs characterized by high positive magnetostriction, rectangular hysteresis loops, as well as high saturation magnetization^18^,^22^ are expectedly desirable for magnetic hyperthermia. Given their finite length and mechanical flexibility, the microwires can be passed through a guide needle into the tumor area, and after the heating treatment, they can be easily extracted as a pin, contrary to the nanoparticles. The low cost of Fe-based AGCMs is an additional advantage. In this research, we report upon the excellent heating properties of Fe_71.7_Si_11_B_13.4_Nb_3_Ni_0.9_ AGCMs.

## Results and Discussion

**Figure 1:**
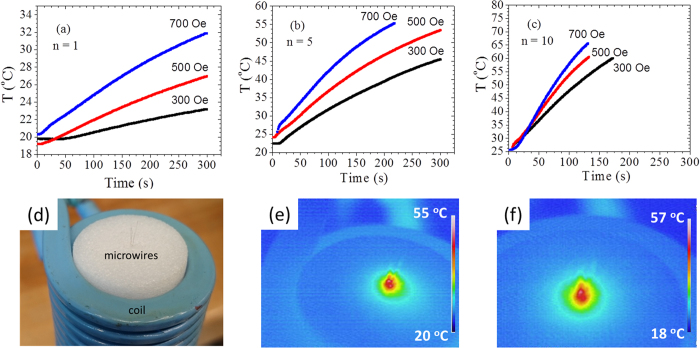
Temperature (*T*) as a function of time (*t*) for 1 microwire (**a**) 5 microwires (**b**) and 10 microwires (**c**) of Fe_71.7_Si_11_B_13.4_Nb_3_Ni_0.9_ at different values of AC field. Photography and infra-red thermal camera images of the microwire during AC hyperthermia (**d**–**f**).

First we have investigated the heating properties of the microwire samples with varying wire-number (*n* =1, 5, and 10). Wire pieces of the same length (5 mm) were used for all the experiments. During the hyperthermia experiments, the temperature evolution as a function of time was recorded while applying different AC fields (Fig. 1a–c). As one can clearly see in this figure, the increase in temperature became faster as we increased either the AC field or the number of wires employed. These indicate that by tuning the AC field amplitude and/or the number of wires, the therapeutic window (40–45 °C) for cancer treatment can be easily reached within a few minutes. For a better depiction of the heat transfer, the temperature rise was also recorded using an infra-red thermal camera, as depicted in Fig. 1d–f. As can be seen, the main temperature rise occurred in the area occupied by the wires, ensuring a localized heating.

**Figure 2:**
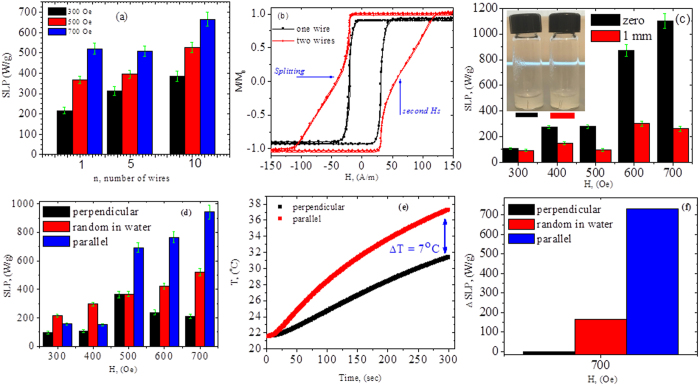
SLP as a function of the number of wires (*n* = 1, 5, and 10) for different AC fields (**a**). AC hysteresis loops measured at a frequency of 115 Hz for one and two wires showing the splitting as a result of different values of switching fields (**b**). SLP as a function of AC field for two microwires separated at the 0 and 1 mm distance (**c**). Inset of Fig. 2c shows the microwires aligned in agar at the 0 and 1 mm distance; SLP as a function of AC field for the microwire aligned, in agar, parallel and perpendicular to the direction of the applied AC field, in comparison with that of the microwire oriented randomly in water (**d**). The temperature (T) as a function of time for two different orientations (**e**) and subtracted SLP values at 700 Oe with respect to the AC field orientation (**f**).

Once the heating curves have been measured, the SLP values are evaluated from their initial slopes (see methods), following the calorimetric method^2^:


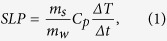


where 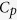 is the specific heat of the solution, *m*_s_ the mass of the solution, *m*_w_ the mass of the microwires, and Δ*T*/δ*t* the initial slope of the heating curves. Fig. 2a shows SLP values of the samples with *n* = 1, 5 and 10 microwires for different AC fields (300, 500, and 700 Oe). It can be observed that SLP increases with increasing AC field for all the samples, as has been typically reported for magnetic nanoparticles^23^. For a single microwire (*n* = 1), SLP increased from 217 W/g for *H* = 300 Oe to 521 W/g for *H* = 700 Oe. These values of SLP are larger than those typically reported for magnetic nanoparticles^1^,^2^ (200–400 W/g) and other magnetic wires of similar composition (~100 W/g)^15^ for similar AC fields and frequencies.

It is interesting to note in Fig. 2a that SLP tended to increase with increasing number of wires. For *H* = 300 Oe, SLP increased from 217 W/g for the *n* = 1 sample to 314 W/g for the *n* = 5 sample and to 385 W/g for the *n* = 10 sample. This suggests that a system composed of multiple Fe-based AGCMs is promising for magnetic hyperthermia. In an interacting multi-wires system, the magnetostatic interaction is often significant, giving rise to the hysteresis loop splitting^24^,^25^. This effect depends mainly on the value of the switching field (*H*_S_) of AGCMs and has been attributed to the superposition of both the external magnetic field and stray field originated by each microwire magnetized in an axial direction^24^,^25^. After switching the magnetization of a wire with lower *H*_*S*_, it produces a stray field in the opposite sense to the external magnetic field, therefore, the total field becomes insufficient to switch the magnetization in the second wire with larger *H*_*S*_ resulting in splitting, thus increasing the hysteresis loop area (see Fig. 2b), which is a favorable prerequisite for rising the hysteresis losses and hence the SLP (Fig. 2a). The aforementioned magnetostatic interaction is in direct connection with the separation distance between two wires, increasing by decreasing the distance between them^25^,^26^. Accordingly, the heating of a multi-wires system should be affected by wires’ separation. To further elucidate this, we have studied the effect of separation between two wires on their heating efficiency. In this study, two identical wire pieces of the same length (5 mm) were embedded in a 2% agar solution as depicted in the inset of Fig. 2c. The wires were separated at (i) zero and (ii) 1 mm for comparison. As one can see clearly in Fig. 2c, SLP decreased as the distance between the wires increased from 0 to 1 mm. The change in SLP became noticeable at high AC fields: the difference in SLP values with respect to the separation distance was 13 W/g for *H* = 300 Oe, while it was 841 W/g for *H* = 700 Oe. The magnetostatic interaction between the wires gave rise to the large SLP of up to 1102 W/g in the presently investigated multi-wires system (Fig. 2c).

On the other hand, shape anisotropy, determined by a longitudinal easy axis in the metallic core due to a particular domain structure^22^,^27^, has been reported to play an important role in the observed magnetic properties of Fe-based AGCMs^18^,^20^. Therefore it is important to understand the effect of wire alignment on the SLP of the presently investigated samples. In this study, magnetic hyperthermia measurements on a single wire subject to two AC field directions (namely, the AC field was applied (i) parallel to and (ii) perpendicular to the length of the wire) were performed and analyzed. In order to obtain the desired wire orientations and eliminate the effect of physical motion on SLP, the wires were immersed in a 2% agar solution. The results obtained are compared to that of the wire immersed in water in a randomly oriented fashion (Fig. 2d). As can be clearly seen in this figure, for the parallel orientation the temperature increased faster than for the perpendicular orientation. By subtracting the heating curve *T*(*t*) for the parallel case from that for the perpendicular case (Fig. 2e), we find a faster temperature change when the wire was aligned along the AC field direction (the parallel case). The change in wire orientation can alter the SLP value by nearly 5 times: at *H* =700 Oe SLP decreased from 942 W/g for the parallel orientation to 210 W/g for the perpendicular orientation (see Fig. 2f). This highlights the important effect of wire alignment on the heating efficiency. This effect was less pronounced at lower AC fields (e.g. 300 Oe) but became significant for higher AC fields (e.g. 700 Oe). When the wire was introduced in water, it tended to slightly align itself with the AC field, but due to the misalignment of the wire with respect to the AC field, the obtained SLP value in water decreased when compared to those aligned in agar (Fig. 2d).

Finally, we have attempted to shed some light on the heating mechanisms associated with the Fe-based AGCMs subject to AC fields. As discussed above, eddy-current loss and magnetic hysteresis loss are two main possible contributions to the heating efficiency^28^,^29^. The heating mechanism related to eddy currents can be explained in terms of the power loss dissipation induced by the change in magnetization, which increases proportionally to the square root of the frequency as long as the flux penetration is complete according to: 
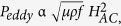
 where *p* is the power lost per unit mass, *μ* is the effective permeability, *ρ* the resistivity, and *f* the frequency of an AC field. On the other hand, the magnetic field produced by eddy current always opposes the change in magnetization, so that the latter is damped away inside the microwire, and if the frequency is sufficiently high, the eddy current losses can become significant. In addition, the skin effect gives rise to an increase in the effective resistance of the metallic core to the passage of large quantities of currents. Therefore it greatly increases the heating efficiency caused by the eddy currents induced into the microwire. In addition, the AC field repeatedly magnetizes and demagnetizes the microwire causing a considerable friction and heating inside the microwire sample. This heating effect is known as a hysteresis loss, which is in a direct correlation with the area of the AC hysteresis loop.

**Figure 3:**
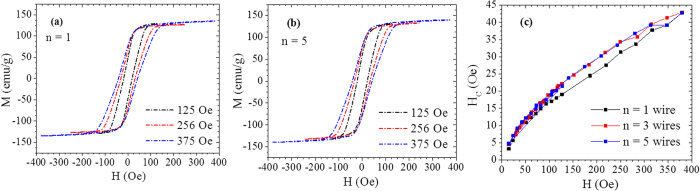
AC hysteresis loops of *n* = 1 Fe_71.7_Si_11_B_13.4_Nb_3_Ni_0.9_ AGCM (**a**) *n* = 5 for different AC magnetic field amplitudes (**b**) and coercive field (*H*_*c*_) as a function of AC field for the *n* = 1, 3 and 5 wire samples (**c**).

We have measured AC hysteresis loops of Fe-based AGCM samples with varying number of wires (see Fig. 3a,b for *n* = 1 and *n* = 5). Magnetic field dependences of the coercive field, *H*_*c*_, deduced from these loops are plotted in Fig. 3c. The values of *H*_*c*_ are larger for the multi-wire systems (*n* = 3 and 5) than for the single wire (*n* = 1), especially for AC fields exceeding 100 Oe (Fig. 3c). This AC coercivity enhancement must be attributed to the important effect of magnetostatic interactions between the wires. In addition, the AC hysteresis loops present a rectangular shape for different field amplitudes (Fig. 3a,b), which is ideal to maximize the hysteresis losses. By increasing the magnetic field amplitude, *H*_*c*_ increased (Fig. 3c) while the saturation magnetization, *M*_*s*_, remained almost the same (Fig. 3a,b), thus increasing the heating efficiency of the wires.

It is generally known that blood perfusion rate is an important factor in all magnetic nanoparticles related biomedical applications that imply indirect intravenous injection of nanoparticles that have to circulate through the blood stream to reach the cancer area. Hence, the heat generated by nanoparticle transfers with the blood flow^30^,^31^. In case of the microwires, however, they can be directly inserted into a tumor area, so this problem can be avoided. In any case, a good understanding of the blood perfusion rate or similar effects warrants a thorough study which is beyond the scope of the present work.

## Conclusions

In summary, we have shown that the Fe-based AGCMs possess considerable heating properties and, in addition to their low-cost fabrication, they are a promising candidate material for prospective applications in magnetic hyperthermia. We have critically scoped that by the use of only single microwire; the shape anisotropy determined by a longitudinal easy axis in the metallic core due to a peculiar domain structure of Fe-based microwires coupled with the parallel configuration at higher applied magnetic field is an optimal profile for the efficient release of heat (942 W/g). In the case of several microwires, the implementation of two nearby samples does not affect negatively the heat efficiency due to a dipole switching and the magnetostatic interaction between the wires gave rise to the large SLP of up to 1102 W/g. Besides, the protective glass-coating layer itself can be considered as an insulating barrier which enhances the biocompatibility of this family of soft ferromagnetic materials for bio-medical applications. Our study opens up new directions for further research into the magnetic hyperthermia based therapy applications of this class of microwire.

## Methods

Fe_71.7_Si_11_B_13.4_Nb_3_Ni_0.9_ AGCMs were fabricated by the modified Taylor-Ulitovsky method as described elsewhere^20^. In short, this method essentially involves the direct casting of the metallic ingot from the melt and consists of a simultaneous drawing of the composite microwire (metallic nucleus inside the glass capillary) through the quenching liquid (water or oil) jet onto a rotating bobbin. Achieved rapid quenching rate allows for the preparation of amorphous microwires^18^,^19^,^20^. Amorphous structure is essential for achievement of improved mechanical properties, such as plasticity and flexibility as well as high tensile yield^19^,^20^.

**Figure 4:**
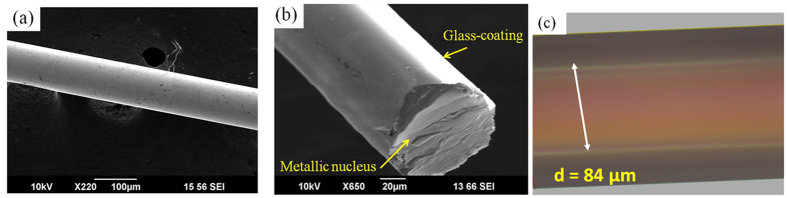
SEM image of a Fe_71.7_Si_11_B_13.4_Nb_3_Ni_0.9_ AGCM (**a**) with a cross-sectional image of the wire (**b**) and optical microscope image in the transmitted light mode showing the metallic nucleus core and glass-coating layer (**c**).

A JEOL JSM-6390LV scanning electron microscope (SEM) was used to examine the wire’s topology (Fig. 4a,b). The metallic nucleus diameter (84 μm) and the glass coating thickness (25 μm) were determined using an Axio Scope A1 optical microscope (see Fig. 4c).

Around room temperature, magnetic hyperthermia experiments were carried out by calorimetric methods using a commercial 4.2 kW Ambrell Easyheat LI 3542 system. The single microwire samples were prepared by inserting a single piece of about 5 mm in length (mass~0.243 mg) into a glass vial filled with 1 mL of deionized water. For wire orientation tests, the single microwire was placed into a 2% agar solution. The orientation was imposed by the use of a small external permanent magnet and physically aligning before the agar cooled completely. The vial together with the microwire samples inside were placed into a coil connected to a power generator, which allows us to control the amplitude of the AC field inside the coil. While the field is applied, a fiber optic temperature sensor inserted into the vial with the solution records the increase in temperature, and from the initial slope of these temperature versus time T(t) curves, one can evaluate the SLP values under the assumption that heat losses are negligible during a certain time interval at the beginning of heating process^32^. A thermal camera was also employed to capture the heat transfer from the microwires.

During these experiments, the frequency was kept at 310 kHz, the AC magnetic field amplitude was tuned from 300-700 Oe. The AC magnetic field amplitude were both measured and calculated, giving a similar AC magnetic field amplitude in either both results. For the calculation of *H* (in Oe), we used the simple formula:


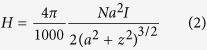


where *N* is the number of turns, *I* is the current (A), *a* is the radius of the coil (m), *z* is the distance from the center (m).

For the experimental determination of the applied AC magnetic field, we used a pick up coil: A pick up coil is a relatively small coil with known surface area and number of turns that is connected to an oscilloscope placed in the middle of the RF coil. The loose wires are twisted around one another to reduce effect of magnetic field. Magnetic field strength, *H*, (Oe) is then regulated by:


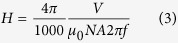


where *V* is the pick to pick volts measured by oscilloscope, *N* the number of turns in the pickup coil, *A* the surface area of the pickup coil (m^2^), *f* the frequency (Hz), and *μ*_0_ is the vacuum permeability. For example, for *H* = 700 Oe, a pick to pick voltage of ~100 V was measured using a pick up coil of 10 turns and 10 mm diameter.

The AC hysteresis loops were measured using a lab-made electromagnetic applicator as described elsewhere^33^. Briefly, it consists of an air-core inductor part of a resonant circuit fed by a power amplifier. The dynamic magnetization, *M*_t_, is obtained by a pick-up coil system composed of two coils wound in opposite direction. The signal is filtered using a low-pass filter with the cutoff frequency at 3 MHz.

## Additional Information

**How to cite this article**: Talaat, A. *et al*. Ferromagnetic glass-coated microwires with good heating properties for magnetic hyperthermia. *Sci. Rep.* **6**, 39300; doi: 10.1038/srep39300 (2016).

**Publisher's note:** Springer Nature remains neutral with regard to jurisdictional claims in published maps and institutional affiliations.
